# Completeness of death registration in Ghana: An evaluation of multiple data sources and methods

**DOI:** 10.1371/journal.pone.0354361

**Published:** 2026-07-23

**Authors:** Charles Agyei-Asabere, Ayaga Agula Bawah, Faustina Frempong-Ainguah, Jill Baumgartner, Samuel Agyei-Mensah, Emmanuel Botchway, Victor Sowah Abordo, Majid Ezzati

**Affiliations:** 1 Regional Institute for Population Studies, University of Ghana, Accra, Ghana; 2 Institute for Health and Social Policy, McGill University, Montreal, Canada; 3 Department of Geography, University of Ghana, Accra, Ghana; 4 Births and Deaths Registry, Accra, Ghana; 5 Faculty of Medicine, Imperial College, London, United Kingdom; Ensign Global College, GHANA

## Abstract

**Introduction:**

The lack of a robust and comprehensive civil registration system undermines efforts to develop policies that address emerging health issues in a population. While Ghana has made significant strides in birth registration, progress in death registration has lagged despite the system’s introduction in 1888. Death records are dispersed across institutions and hampered by weak coordination and inconsistent data sharing. The study aims to evaluate the completeness of death registration using both demographic and empirical methods.

**Methods:**

We assessed the quality of mortality data using demographic methods and further applied Death Distribution Methods (DDM) as well as the Empirical Completeness Method (ECM) to evaluate the completeness of death registration from the 2022 mortality register and two rounds of population and housing censuses.

**Findings:**

Age reporting ranged between fairly accurate and rough, with noticeable under-reporting of under-five deaths. Death registration completeness was estimated at 28.7 percent and 56 percent using the ECM and DDM, respectively. DDM results reveal that male completeness was highest (101%) in the Volta Region and lowest (42%) in the Western Region, while female completeness was highest in Upper East (89%) and lowest in Western (35%) regions. Sub-national variations in completeness were further highlighted by the ECM, with completeness ranging from nearly 70 percent in Greater Accra to barely 8 percent in the Oti Region.

**Conclusions:**

The study revealed that completeness of death registration remains low and incomplete, with minimal improvement over the past two decades. The findings underscore the urgent need to adopt innovative, targeted and coordinated approaches to improve completeness, ensuring Ghana can meet international commitments such as SDG 17.19.2.

## Introduction

Death registration plays a key role in the production of accurate, timely, and reliable information on mortality and its causes, which is essential for developing effective public health policies to address emerging health issues in any population [1–4]. This aligns with global (the 2030 Agenda for Sustainable Development), regional (Agenda 2063: The Africa We Want), and national (Agenda for Jobs II: Creating Prosperity and Equal Opportunity for All 2022–2025) development goals. As reducing under-five and maternal mortality, as well as mortality rates attributable to non-communicable diseases, has become a focus, it is essential that countries are able to measure completely and accurately all forms of death to help monitor these targets. Although most countries have a method of recording deaths, completeness varies, with highly developed countries often achieving near completeness of registration [5–7].

Despite adopting successful methods, many low and middle-income countries (LMIC), especially in sub-Saharan Africa (SSA), struggle to achieve adequate completeness of death registration [8]. While improvements in healthcare and education often correlate with better registration, examples from Mexico, Serbia, and Moldova show that national wealth alone does not ensure registration [7,9,10]. Individual factors such as age, sex, socioeconomic status, and the deceased’s place of residence [11,12], circumstances of death, and where the death occurred [13] may influence the chances of the event being registered. Beyond these, the Civil Registration System (CRS) serves as a systemic determinant of death registration completeness [14]. Implying, not every death has the same chance of being registered.

The need to accurately record the number of births, deaths, and their causes has driven global, regional, and national efforts to strengthen the Civil Registration and Vital Statistics (CRVS) systems [15,16]. For instance, the Sustainable Development Goal (SGD) indicator 17.19.2 emphasizes the need for countries to achieve 100 percent birth registration and 80 percent death registration by 2030 [17]. In Ghana, policies such as the requirement of a birth certificate for school admission, issuance of a passport, and the Ghana card have led to improvements in birth registration, with an estimated 74.5 percent coverage of children under-five years [18]. By comparison, death registration occurs at a much lower rate, even though it is required to provide a legal basis for claiming property, social welfare benefits, remarriage, and the transportation of deceased persons across administrative boundaries [11].

The SDG theme of “leaving no one behind” aligns with Ghana’s development goal of ensuring a good and equitable national health system. To achieve these goals, there is a need for accurate, reliable, and timely data on deaths by cause and location, as well as other socio-demographic characteristics such as age, sex, and socioeconomic status. These are critical indicators that aid health policymakers and implementers identify health threats among the most vulnerable groups. Some of these targets and indicators depend on harnessing information from administrative records of death registration, a service that the civil registration system can best provide [19] compared to censuses and surveys.

Ghana has a long history of death registration, dating back to 1888 as the Cemeteries Ordinance, under British colonial rule. The law was enacted as the Birth and Death Registration Act, Act 301 in 1965, and amended in 2020 as the Registration of Births and Deaths Act 1027. The Act stipulates that all births and deaths (including foetal deaths) must be registered and that a certificate must be issued. It prohibits burial without a death certificate. Despite its significance and legal mandate, the registration of death is low in Ghana [14,20].

A contributory factor to the low death registration in Ghana is the 10-day period allowed for free registration after an occurrence [21]. Additionally, socio-cultural emphasis on longevity and celebration of death at old age (typically 70 years and above), increasing the likelihood of registering deaths among the elderly rather than among younger age groups [13,22]. In contrast, deaths of younger people and those from unnatural causes are considered “bad deaths” and are likely to go unrecorded and unregistered, particularly if they occur outside of health facilities or in remote areas where limited access to infrastructure and registration services discourages death registration [20]. Employment-related incentives also shape the death registration dynamics: deaths among workers in the formal sector are likely to have their deaths registered because of the requirement of a death certificate to access pensions and estate claims. By contrast, deaths of informal or unemployed workers, who may not have such social security entitlements, are less likely to prompt registration [20]. In addition, religious and cultural practices influence the likelihood that a death will be registered. Among Ghanian Muslim communities, Islamic tradition requires burial within 24 hours of death, making it difficult to obtain a death certificate prior to burial, reducing the chances of a formal death registration [22].

Administrative records on death are not centralised but collected by various formal and informal institutions, including the Births and Deaths Registry (BDR), health institutions (including BDR units now embedded in major hospitals where death certificates are issued at the point of care), surveillance sites, the police (coroner’s report), religious organizations, funeral homes, and cemeteries. However, these records are dispersed and often operate independently [14]. Consequently, none of these sources offers a comprehensive overview of population mortality statistics, which are susceptible to both undercounting and double-counting, leading to inaccuracies. These challenges make it difficult to estimate the completeness of death data from the available empirical sources, hence the national statistics office relies on surveys and censuses to estimate population deaths.

In addition, the completeness of death registration at the subnational level in Ghana is poorly understood and researched, with only studies from 1999, 2007, and 2024 [23–25] offering limited insights for the subnational level. Presently, it is unclear whether the completeness of death registration has improved over the last three decades. This lack of detailed data, particularly for other administrative regions, is exacerbated by methodological challenges, data access issues, and reliance on decennial census data. Inequalities in death reporting are likely in Ghana, given the varied cultural practices, distances to registration centers, poverty levels, urbanization, and health facility distribution.

The BDR plays a crucial role in vital statistics collection in Ghana, and its recent report estimated death registration completeness at 37.8 percent [25]. However, a critical gap exists in evaluating this estimate within the broader understanding of national death registration completeness needed for effective policy and planning. Given the importance of accurate mortality statistics and the reportedly low completeness of death registration, this study evaluates death registration completeness in Ghana using multiple sources and estimation methods.

## Data and methods

### Data

Ghana’s Registration of Births and Deaths Act (Act 1027) has three main objectives: a) to promote public health, b) extend registration to facilities across Ghana and establish an efficient system of recording births and deaths, and c) to obtain vital statistics that are adequate and efficient to provide reliable estimates for public health planning. While Ghana has several independent mortality information subsystems (S2 Table), most operate in silos rather than feeding into the central BDR mortality register, as ideally intended [14]. The two primary sources of annual mortality data are the BDR’s administrative Mortality Register (MR) and the Population and Housing Census (PHC), conducted approximately every ten years by the Ghana Statistical Service (GSS) through surveys (Table 1).

**Table 1:** Sources of mortality information.

Name	Year	Age	Source	Comment
PHC	2010	Single and 5-year age groups	GSS	Published report and micro data
PHC	2021	Single and 5-year age groups	GSS	Published report and micro data
Mortality register	2022	5-year age groups	BDR	Published report

For this study, age and sex summaries of deaths were obtained from the published 2022 BDR reports [25] and 2010/2021 PHC reports [26]. Regional-level population counts (denominator) were sourced from the STATSBANK online portal hosted by GSS, with additional data provided upon official request. To further enrich the mortality data landscape, the PHC included questions about household deaths by age and sex that occurred within the 12 months preceding the census. However, because of the nature of censuses, they did not include follow-up questions to determine whether the reported deaths had undergone official registration.

All data, whether obtained from administrative records, censuses, or surveys, are subject to errors. The extent of the errors may depend on how accurately the information was recorded and the degree of coverage of the study area. The most pronounced errors are related to age. To generate an accurate and reliable estimation of death registration from multiple sources, it is important to evaluate these to determine the accuracy of the information. The first stage of the analysis focuses on the quality of age information, which is evaluated using age heaping metrics applicable to the micro data sourced from the censuses.

### Methods

#### Age data evaluation.

The Whipple and Myers Indices are age heaping metrics assumed to provide a fair measure of the reliability of age distribution in a population. These were employed to assess the quality of age data, as age structure is important in estimating mortality. Age records from censuses and surveys are reported by household members and other non-related members, which can lead to some inaccuracies in age data. These measures assess the age distribution and identify unusual patterns, including avoidance and preference for terminal digits [27].

#### Estimating completeness of death registration.

To assess the completeness of death registration in the study area, the quality of mortality data from both sources was evaluated using the Death Distribution Methods (DDM) and Empirical Completeness Method (ECM).

**Death Distribution Methods (DDM):** The Death Distribution Methods (DDM) assess the completeness of death registration and the consistency of age patterns on reported deaths in a population, while also assessing the relative completeness of one census in relation to another. These methods are suitable when underlying assumptions, such as a closed population (or known net migration) and constant age-specific death rates during the intercensal period, are met, and they are used in contexts where there are no effective civil registration systems. In this approach, we use reported household deaths, backed by the Act 1027, which states that all deaths must be registered as a proxy measure of potential deaths registered. There are three main approaches to estimating completeness of death registration and the coverage of population and death recordings: the Generalized Growth Balance method (GGB), the Synthetic Extinct Generation method (SEG), and the Hybrid of the two methods (GGB-SEG). It is assumed that age reporting is accurate across all ages of the population, and net migration is known or negligible [28–30].

These methods were applied to the population age distributions from the 2010 and 2021 censuses, as well as the age distributions of the deaths reported in both censuses, analyzing males and females separately.

The GGB utilised the age-sex distribution of the population at the two census points, along with the number of deaths reported, while holding the assumption that the population is closed to migration and follows the general balancing equation. The method is based on the balancing equation given by:


$$\begin{array}{c} \left(a+)=b(a+)-d(a+\right) \end{array}$$

Where (a+) represents an open-ended age segment (all persons aged a and older), r(a+) is the segment-specific growth rate, b(a+) is the entry rate (the rate at which people age into the segment by reaching age a), and d(a+) is the death rate of the segment. That is, the growth rate of any age segment equals the rate of entries into that segment minus the death rate within it [31].

The Synthetic Extinct Generations (SEG) method, on the other hand, is based on the assumption that the observed number of people alive at a given age at any point in time must equal the number of people who will die from that age forward. This approach primarily involves comparing the projected number of deaths in a future cohort with the cohort’s current population size, serving as a measure to evaluate the extent of completeness in the registration of deaths between census periods. The SEG method also relies on the assumption that the completeness of death registration remains unchanged between the two censuses.

The hybrid “GGB-SEG” method combines the strengths of both the GGB and SEG approaches [28]. This involves first applying the GGB method to the available data to adjust for intercensal population growth and coverage differences, followed by the application of the SEG method to the GGB-adjusted population estimates. The hybrid “GGB-SEG” method uses an age trim distribution of 50–70 years (GGB = 40–70 while SEG = 50–80), and has been shown to provide more reliable estimates [29]. These recommended age ranges were derived from a simulation analysis conducted across countries with varying levels of census and death registration quality. Murray and colleagues [29] assessed the sensitivity of completeness estimates to different age trims. Their findings showed that the hybrid method using the 50–70 age range minimizes bias arising from age misreporting, migration, and age-specific coverage errors. Hence, producing more stable completeness estimates.

The DDM is not without limitations. The assumption about population being closed and no net migration may hold for national populations, but not entirely true at the subnational level [28]. In addition, the estimates derived from these are not timely as they rely on two censuses. These limitations, notwithstanding, provide good estimates of the completeness of death registration at the national and regional levels in the absence of reliable CRS data.

Drawing on population and death counts from the 2010 and 2021 Ghana population and housing censuses, the three DDM methods were used to evaluate the relative completeness of death registration using the R-package ‘DDM’ developed by Riffe and colleagues [32].

**Empirical completeness method:** The Empirical Completeness Method (ECM) developed by Adair and Lopez [33] was used to estimate the completeness of death registration in the CRS. Unlike the DDM, which often requires data from two successive censuses, the ECM allows flexibility and can be applied to civil registration data, even at lower levels of administration. There are two variants of the ECM: the first takes into consideration the completeness of child (under five years) mortality, while the second does not. The model requires total registered deaths, mid-year population estimate, the proportion of the population aged 65 and above, estimates of under-five mortality, and the registered under-five mortality rate (where applicable). In this study, the second variant of the model was used due to low reporting of under-five deaths in the mortality register, and because registration of child deaths is not expected to be associated with completeness of death registration [33]. Nevertheless, the results for both models have been provided in the supplementary appendix (S1 Table). A variant of the second model is written as follows:


$$\begin{array}{c} \text{logit}\left({\text{C}}_{\text{jk}}^{\text{All}}\right)=\beta_{\text{0}}+{\text{RegCDRsq}}_{\text{jk}}\times \beta_{\text{1}}+{\text{RegCDR}}_{\text{jk}}\times \beta_{\text{2}}+{\%\text{65}}_{\text{jk}}\times \beta_{\text{3}}+{\text{l}\text{n}(\text{5q0})}_{\text{jk}}\times \beta_{\text{4}}+{\text{C}}_{\text{jk}}^{\text{5q0}}\times \beta_{\text{5}}+\text{k}\times \beta_{\text{6}}+{\text{e}}_{\text{jk}}\;+\gamma_{\text{j}} \end{array}$$

The registered crude death rate (RegCDR) was estimated using the number of registered deaths for each region divided by the mid-year population, using the PHC 2021 as the base population. The proportion of the population aged 65 and above (%65+) was estimated by dividing the population aged 65 and above by the mid-year population. Under-five mortality (5q0) rates for Ghana and the 16 regions were estimated from the 2022 Ghana Demographic and Health Survey (GDHS) using the “DHS.rates” R package [34]. Population and death counts (census) and registered deaths (mortality register) were extracted and calculated from published reports by GSS and BDR [25,26].

The ECM has some advantages over DDM, as it does not rely on the assumptions of the DDM; rather, it models completeness on key drivers of mortality in a population and can be readily applied to monitor changes in the completeness of the registration system over time [33,35]. The model has been applied in various global settings, including India, Nepal, China, Egypt, and Ecuador [9,36–39], demonstrating its versatility across diverse populations and data environments. Estimating completeness using both the DDM and ECM methods, along with survey and administrative records, provides a comprehensive understanding of death registration completeness in Ghana. This will support the development of strategies to improve the accuracy and reliability of vital statistics in the region.

### Assumptions

For the methods used to estimate the completeness of death registration, based on the population age distribution of the 2010 and 2021 PHC and the deaths recorded during the study period, the death distribution methods in this study make three assumptions: a) the population is closed to migration; b) birth rates and death rates are constant; and c) the extent of age misreporting and other errors are minimal [29]. While there are no data to measure net migration, the study assumes that net migration in the population is negligible [40].

## Results

Table 2 shows the number of reported deaths in Ghana based on the PHC 2010 and 2021, and registered deaths from the 2022 mortality register. The 2010 and 2021 PHCs recorded a total of 163,534 and 132,199 deaths respectively, while the mortality register recorded 50,992 deaths for the year 2022. In all data sources, more male deaths were recorded than female deaths, with almost 58 percent of deaths in 2021 being male. The discrepancy between the two data sources (census and mortality register) could be that the census is a complete enumeration of vital events (deaths) within the last 12 months, whereas the mortality register in Ghana operates passively, waiting for informants to declare deaths and complete administrative processes to register a death [14,41].

**Table 2:** Reported deaths by year of occurrence.

Year	Source	Males	Females	Total
2010	PHC	84,214	79,320	163,534
2021	PHC	76,067	56,132	132,199
2022	BDR	27,907	23,085	50,992

### Evaluation of mortality data from 2010, 2021, and 2022

**Fig 1:**
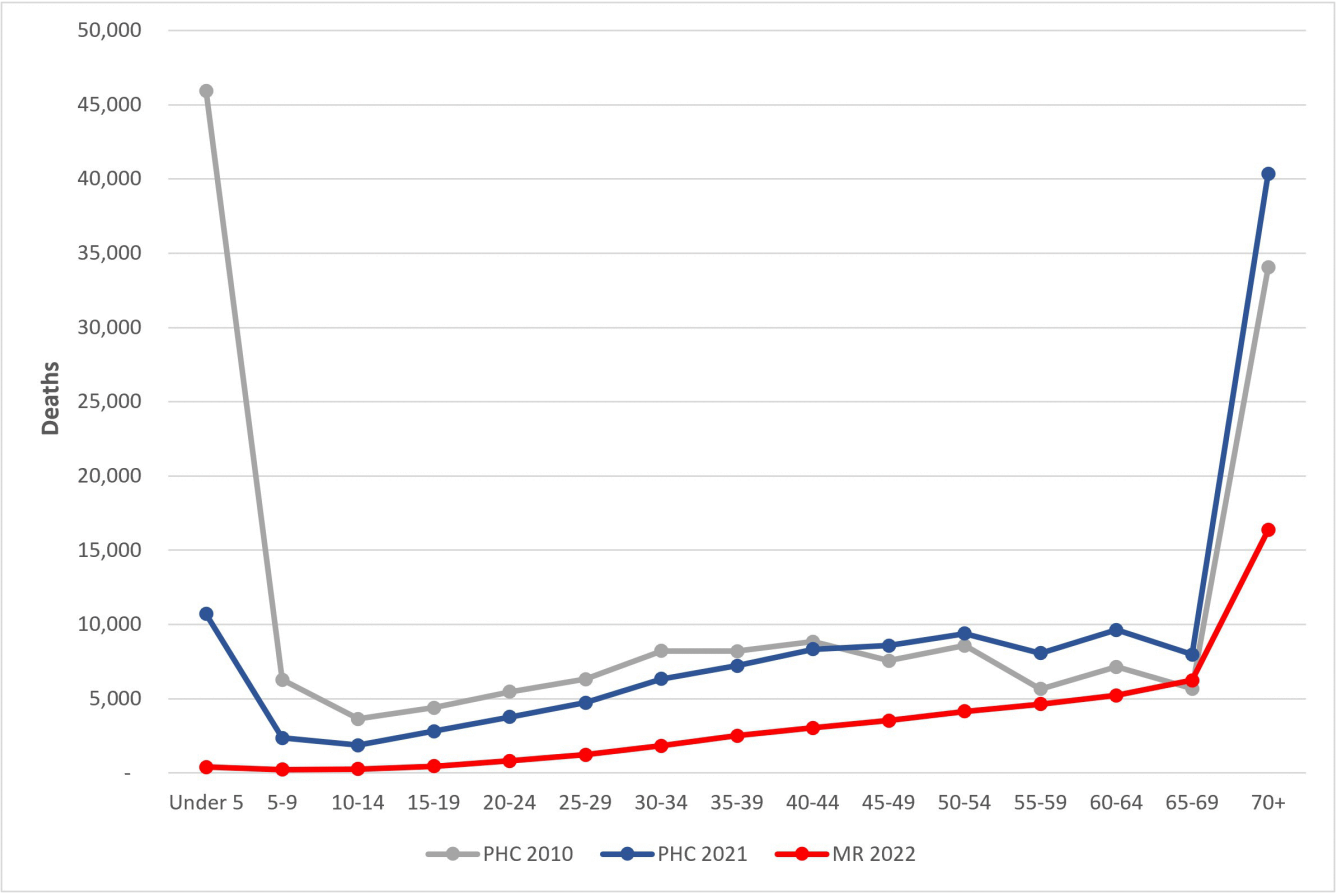
Comparison of mortality data by age group (PHC 2010, 2021; and MR 2021).

Fig 1, shows the reported age at death from the censuses and the mortality register. The censuses reported significantly higher deaths than the mortality register, underscoring substantial underreporting of deaths in the mortality register, especially under-five deaths. Comparing the 2010 and 2021 censuses, there was a noticeable reduction in under-five deaths, which could reflect improvements in child survival [42] and overall population health, with a substantially higher number of deaths happening at older ages (65–69) in 2021 relative to 2010. However, the 407 under-five deaths captured in the MR in 2022, compared to the 10,717 deaths in the 2021 census, reflect the mortality registration system’s failure to capture infant and child deaths. Deaths reported in the MR generally increase with age from 20–24–65–69 years, likely due to administrative requirements for accessing social welfare schemes such as deceased pensions or estates [20]. This trend continues, with the highest number of deaths reported in the age group 70+ in the MR, further suggesting that older individuals are more likely to be registered than younger ones [13]. Despite the age-related pattern in death registration, the overall reporting in the MR remains low when compared to death counts in both censuses.

In Table 3, using the PHC data, Whipple’s index for reported population ages (23–62) was observed to be approximate for both males and females. However, for both sexes, there have been improvements in the quality of age reporting between 2010 and 2021. With respect to reported deaths, a Whipple’s index of 151.4 was observed for males and 149.3 for females in the year 2010, while in 2021, the age evaluation was 137.8 for males and 134.4 for females.

**Table 3:** Data quality assessment of Ghana PHC population and reported deaths 2010 and 2021.

	Data source	Year	Male		Female	
			Whipple	Myers	Whipple	Myers
Population	Census	2010	123.7	5.4	124.4	5.7
	Census	2021	111.4	3.3	109.6	2.9
Deaths	Census	2010	151.4	13.8	149.3	13.5
	Census	2021	137.8	9.3	134.4	8.8

### Completeness of death registration

For the period between 2010 and 2021, death registration relative to population count was notably low and reflects a regional and gender gap. Using the GGB-SEG method, males (56.8%) have a slightly higher registration completeness than females (56.2%) at the national level (Table 4). While the national average is nearly equal for both males and females, there are sub-national and gender disparities. The highest registration completeness was observed in the Volta region for males, while the least completeness was observed in the Western Region for both males and females. For instance, there is male-dominant completeness in regions such as Volta (101.2% male vs. 67.8% female), Upper West (82.2% male vs. 75.3% female), Central (70.5% male vs. 55.4% female), and Bono (67.8% male vs 48.3% female). Interestingly, in the northern-belt, specifically Upper East (86.9% male vs. 89.0% female), North East (47.9% male vs. 77.1% female), Northern (47.7% male vs 68.8% female), and Savannah (44.0% male vs. 53.3% female), female registration completeness significantly exceeds male registration completeness.

**Table 4:** Completeness of death registration in Ghana, 2010 and 2021 census using DDMs.

Area	Males			Female		
	GGB	SEG	GGB-SEG	GGB	SEG	GGB-SEG
Ghana	51.4	63.4	56.8	61.7	48.5	56.2
Western	33.4	49.2	42.3	32.7	32.7	35.2
Central	61.3	97.8	70.5	47.6	65.6	55.4
Greater Accra	31.8	56.1	46.5	27.1	42.7	38.2
Volta	98.6	98.6	101.2	71.8	58.7	67.8
Eastern	69.9	66.6	66.6	66.8	52.7	61.6
Ashanti	58.2	46.0	54.5	69.5	37.5	58.4
Western North	37.0	49.3	44.4	59.6	42.9	52.5
Ahafo	46.7	48.9	48.2	57.1	42.1	51.3
Bono	61.6	85.3	67.8	44.9	53.2	48.3
Bono East	35.5	55.4	46.3	54.4	45.3	49.0
Oti	45.4	43.2	45.1	55.6	37.8	47.4
Northern	35.7	57.8	47.7	78.3	53.3	68.8
Savannah	29.3	56.0	44.0	71.0	36.1	53.3
North East	40.9	55.1	47.9	99.7	48.4	77.1
Upper East	84.7	107.2	86.9	105.4	75.4	89.0
Upper West	78.6	101.6	82.2	90.2	62.4	75.3

Source: Estimated from Ghana population and housing censuses.

**Fig 2:**
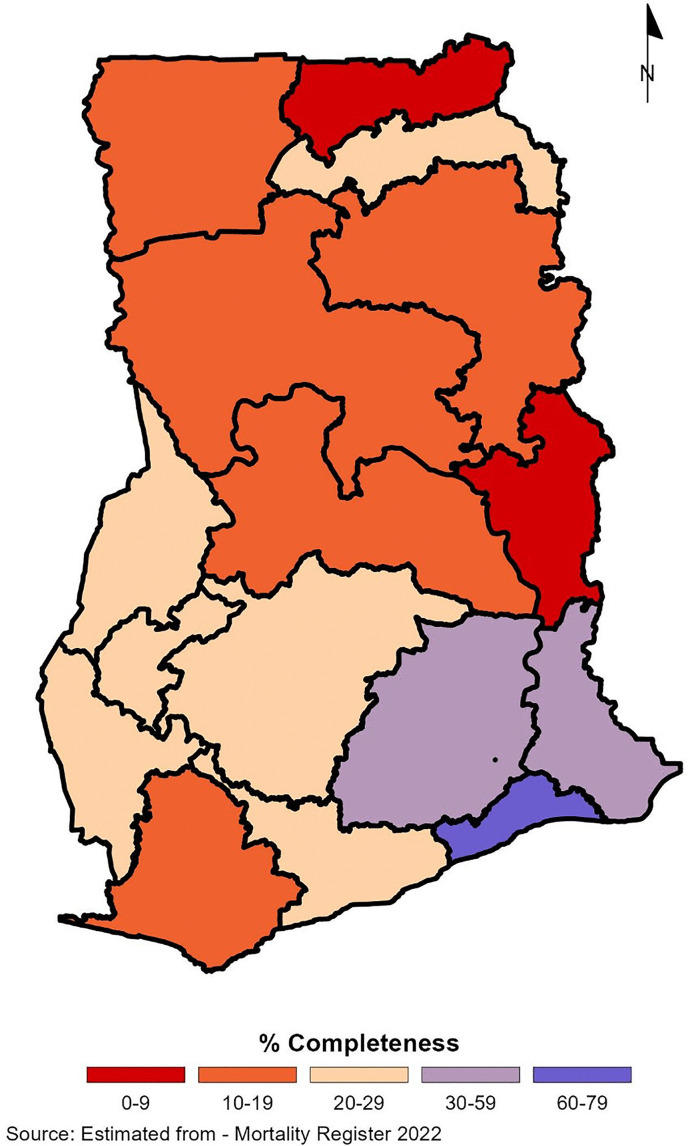
Map of Ghana showing completeness of death registration.

Using the ECM on the MR, death registration in Ghana was estimated to be very low, with 28.7 percent completeness (Table 5). At the subnational level, completeness varied from 7.6 percent in the Oti region to about 70 percent in the Greater Accra region. From Fig 2, it was observed that regions in the southeast of Ghana, such as Eastern (39%), Volta (47.1%), and Greater Accra (69.8%) regions had some appreciable levels of completeness, though generally low. Most of the regions with less than 20 percent completeness were located in the periphery of Ghana. It is not surprising that Greater Accra exhibits the highest level of completeness.

**Table 5:** Completeness (%) of death registration in 2022 using the mortality register – Empirical Completeness Method.

Area	Both sexes	Male	Female
Ghana	28.7	28.9	31.6
Western	17.1	18.1	18.6
Central	21.5	18.0	31.2
Greater Accra	69.8	62.0	81.0
Volta	47.1	52.9	43.9
Eastern	39.0	40.6	39.9
Ashanti	29.9	34.4	28.1
Western North	25.3	18.6	45.0
Ahafo	29.3	33.5	29.0
Bono	29.8	25.0	44.0
Bono East	12.3	13.6	13.4
Oti	7.6	7.5	9.6
Northern	16.3	18.6	17.3
Savannah	11.1	10.8	14.1
North East	22.8	59.7	24.9
Upper East	9.2	13.7	6.8
Upper West	13.1	11.4	23.0

**Fig 3:**
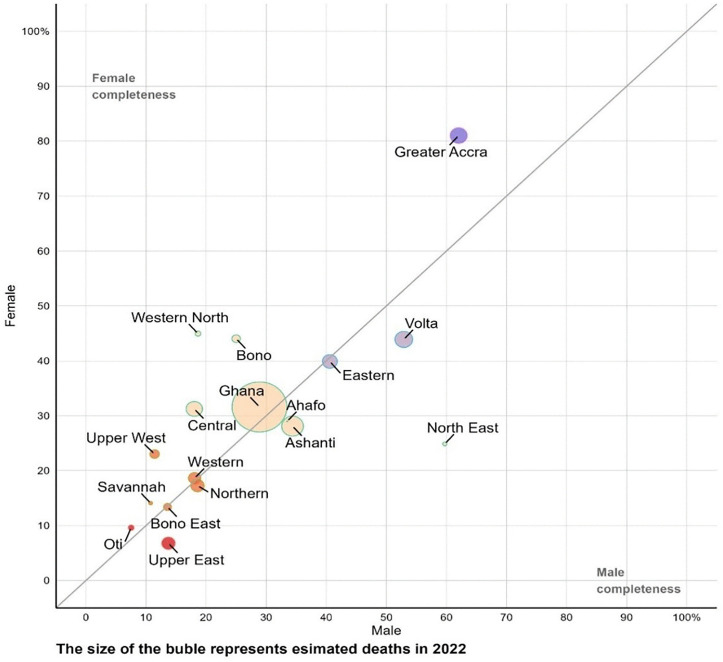
Completeness of death registration by sex and region in Ghana.

Fig 3 shows the distribution of completeness by sex and region from the MR. Female death registration was 2.7 percentage points (p.p) higher than males (31.6% vs 28.9%). When disaggregated by region, female death registration was higher in half of the regions, namely, Greater Accra, Western North, Bono, Central, Upper West, Western, Savannah, and Oti. Specifically, in the Greater Accra region, female registration was 19 p.p. higher than male registration (81% vs 62%), while in the North East region, female death registration was 34 p.p. lower than male death registration (24.9% vs 59.7%). In the Western (18%) and Bono East (13%) regions, about equal proportions of deaths were captured.

## Discussion

The study examined the completeness of death reporting in Ghana and its regions using administrative and census data.

The Evaluation of age data from the 2010 and 2021 population censuses suggests that the quality of population age reporting in Ghana has improved. Evidence of preference for ages ending in 0 and 5 appears to have declined, consistent with findings from Demographic and Health Surveys (DHS) and Multiple Indicator Cluster Surveys (MICS) [43,44]. These improvements may reflect broader socioeconomic development as well as advances in data collection practices, such as the introduction of Computer Assisted Personal Interviewing (CAPI) during the 2021 census.

Despite these gains in population data, substantial age misreporting persists in the mortality data. The Whipple’s Index for reported deaths in 2010 was ‘rough’ for both males (151.4) and females (149.3), and although modest improvements were observed in 2021 (137.8 for males and 134.4 for females), the index remains high, indicating significant age heaping. Such misreporting is common in settings with weak CRVS systems, where deaths are often reported by proxy respondents who may not accurately recall ages or lack documentary verification [45–47]. Continued reliance on proxy reporting and the absence of reliable age documentation can distort age-specific mortality estimates.

This study found completeness of death registration in Ghana to be generally low, with 28.7 percent (28.9% for males and 31.6% for females), and 57 percent (males) and 56 percent (females) using the ECM and DDM, respectively. While the DDM provided higher death registration relative to population count, from this assessment, the ECM is suitable for estimating completeness of death registration in a setting such as Ghana, as it can be used when data are available for only one year and does not mainly rely on intercensal data.

The results indicate sub-optimal completeness of death registration in Ghana when compared with the globally accepted level of 80 percent. The national-level estimate of 28.7 percent shows only marginal improvement relative to earlier assessments of death registration completeness in Ghana. For instance, the United Nations Statistics Division (UNSD) reported a national completeness of 25 percent as of 2014, although this estimate was published in 2023 [5]. Moreover, there has been limited progress since the expert review by Mitala [48], which placed national completeness at approximately 16 percent in 2018. Similarly, at the subnational level, the findings for Greater Accra align closely with earlier studies, such as Kwankye et al. [23], who reported a completeness of 41 percent based on the 2000 census data. It is also noteworthy, though based on data nearly 30 years old, that Stephens et al. [24] estimated 75 percent completeness of the region using administrative data. Collectively, these historical and contemporary comparisons underscore that, despite the incremental gains, death registration in Ghana remains low.

During the COVID-19 pandemic, many countries experienced disruptions in their civil registration systems [49]. In Ghana, during the March-July 2020 lockdown, particularly in the Greater Accra and Ashanti regions [50]. The COVID-19 pandemic introduced additional challenges to an already struggling death registration system. Restrictions on mobility and bans on social gatherings – including funerals (which are key moments for death notifications)- likely reduced the reporting of deaths, even in regions with relatively higher registration completeness. Although the impact of the restrictions appears marginal, relative to pre-existing structural deficits, reduced access to public registration offices and the temporary suspension of community-based reporting mechanisms hindered essential service delivery [51], with repercussions likely extending to the 2022 death reporting.

Comparing documented deaths in the MR 2022 to reported deaths from the PHC 2021, there was gross under-reporting, particularly under-five deaths, with more deaths being reported in the census than the mortality register. This observation is similar to what was observed by Stephens and colleagues [24], who estimated that about 50 percent of under-five deaths were not reported. In a recent study in five African countries, including Ghana, less than 2 percent of neonatal deaths were registered [52]. Although the census has a wider coverage, the low number of deaths recorded for the under-five population in the MR could also be a result of health personnel’s failure to document facility stillbirths or adverse neonatal events for fear of blame and further investigation [53]. From a socio-cultural context, in Ghana and other sub-Saharan African countries, bereaved mothers are advised not to mourn the death of a child as it is seen as a potential source of sterility, a bad omen, and an invitation for a recurrence of the event. As such, they are advised to conceal pregnancy loss [54,55]. Such cultural beliefs and practices, where greater importance is placed on reporting deaths of older people, coupled with a passive registration system practiced in Ghana, lead to low death reporting for younger ages [11].

Comparing deaths reported in the mortality register with the Ghana Health Service (GHS) District Health Management Information System (DHMIS II), Owusu et al. [56] found 14,860 and 24,714 institutional deaths for the years 2017 and 2018, respectively. This is at variance with the number of reported deaths recorded in the mortality register for the same period 2017, and 2018 (48,648 and 49,629, respectively) [25]. In this respect, the mortality register was found to collect more death data than the DHIMSII, as the DHIMSII is facility-based and does not capture community deaths.

A significant obstacle to Ghana’s establishment of a comprehensive death registration system is the fragmentation of its mortality data collection landscape [14,57]. The existence of 16 mortality subsystems with disparate reporting standards and insufficient digital interoperability with the central registry results in significant data leakage and prevents the production of a comprehensive overview of mortality statistics. This fragmentation of mortality data systems has also been documented elsewhere in Peru [14] and Pakistan [58].

With respect to the completeness of registration in the census data, there was not much difference in completeness between the sexes at the national level using the DDM. While there was more male death registration in absolute terms, the empirical completeness method indicated higher female completeness than males using the MR. This is similar to what was observed in Egypt [37], although in different proportions.

These findings are useful in measuring national efforts to improve the CRVS (death registration) to meet international targets such as SDG 17.19.2. It will also aid in generating primary data for routine mortality statistics, which is important in assessing population health, improving life expectancy, and reducing maternal and child mortality [9,59]. It also serves as a viable data source for monitoring progress towards the attainment of some of the SDGs [60].

The relatively high completeness of death registration observed in the Greater Accra Region, as indicated by the MR, can be largely attributed to its highly urbanized setting and the effective enforcement of burial regulations. In the Greater Accra Region, both non-governmental and governmental agencies, such as the Mortuaries and Funeral Facilities Agency (MoFFA), Accra Metropolitan Assembly (AMA), and its jurisdictional local governments, have established systems to ensure that deaths are officially registered before burial, with strict adherence to local bylaws requiring burial permits [20]. Regular monitoring and structured administrative oversight—particularly in metropolitan areas such as Accra, Ho, Koforidua, and Kumasi—have strengthened compliance with these regulations [61].

The Greater Accra region, being the administrative capital and over 95 percent urbanized, benefits from a higher socioeconomic status, better access to health facilities and services, and a greater concentration of pathologists [62]. Per the Registration of Births and Deaths Act, 2022 (Act 1027), all deaths (including community deaths) require a Medical Certificate of Cause of Death (MCCD) for registration. Pathologists who typically issue these certificates are predominantly located in the Greater Accra Region. Consequently, families in other regions may be less willing to navigate the complexities in registering a death, especially community deaths requiring a coroner’s case and potentially incurring fees for an MCCD [20,22,63].

In contrast, other regions face significant challenges that contribute to lower levels of death registration. Weak enforcement mechanisms, higher proportions of rural areas, little or no registration centres, and limited monitoring capacity make it difficult for authorities to ensure that all deaths are officially recorded. In some cases, informal or unregulated burial practices further undermine the integrity of the registration system. This underscores the importance of sensitization of death registration in these areas. The Births and Deaths Registry continues to facilitate registration of births and deaths through the community population register programme, computerization of births and deaths reporting, and opening of additional registration centres in rural communities [64]. There has been a disproportionate increase in birth registration compared to death registration. The grace period for free death registration, currently 14 days, should be extended, as it is for birth registration (365 days), and incentives should be given to informants to motivate registration of death events.

Civil registration systems are made up of several stages that constitute a coherent process involving different government agencies handling different stages of vital event registration, such as reporting (health facilities, police service, MOFFA, and local government), and data capture [65]. As such, multiple approaches involving inter-institutional collaboration from different arms of government, non-governmental institutions, and the general public need to be adopted. If death reporting is to be improved, then health-seeking and death reporting at the health facility level must be improved.

According to the World Health Organization (WHO) recommendation, there should be a system to report household deaths [66]. The Community Health Planning and Services (CHPS) model, which has proven to be an effective system for health service delivery at the locality level [67–70], could be leveraged to facilitate death reporting and registration. Deaths occurring in communities can be reported, and causes of death determined through verbal autopsy and the information passed on to BDR for registration, compared to deaths that occur at the community-level and are never recorded [71].

Deaths that occur within communities but outside health facilities can be captured using a network of community key informants, i.e., political or religious (e.g., chiefs, assemblymen, Imams, pastors), some of whom are power brokers in their communities. Community informants have a firmer grasp of information on vital events in localities than registration officers. To achieve results through collaboration, these key informants can be tasked with documenting vital events that occur at home within their jurisdictions. Especially in places where there are no BDR offices, community stakeholders can leverage the use of mobile networks and digital platforms [66] to transmit death events to BDR.

Finally, there has been a push for innovative ideas to find the missing deaths. A rich non-traditional resource that has been underutlilised is the obituary system, which is used to announce a death and inform residents of a funeral. Most of these deaths are being reported through funeral posters and billboards [72] and on funeral platforms on social media. In addition to the institutional collaboration to improve death registration, these resources should be leveraged by exploring the possibility of extracting death information from obituaries. To avoid double-counting of deaths that are already registered, vital information such as name, age, and place of death can be compared; if the death is not registered. The Births and Deaths Registry, as the mandated authority for civil registration, would be best placed to lead and coordinate the cross-referencing of obituary data with existing death records. Ghana is moving toward a comprehensive national identification system, which will allow officials to match information received with that extracted from the obituary system. This information may capture deaths known at the locality level but not formally registered.

Complementing these non-traditional sources, several innovative, tailored digital approaches have been implemented in Uganda, Bangladesh, Colombia, Myanmar, and Papua New Guinea, in which mobile phones and electronic tablets were provided to personnel or community volunteers to report basic information about community death events via SMS text message. Follow-ups, including verbal autopsies, are then conducted to determine the cause of death [41]. Since their inception, these approaches have led to noticeable improvements in the completeness of death registration and notification.

## Conclusion

Civil registration and vital statistics system is the gold standard for estimating death registration completeness. However, many sub-Saharan African countries including Ghana, do not have a fully functional civil registration and vital statistics system. In light of the data gaps and accessibility issues, we used death counts from censuses as well as registered deaths to evaluate the completeness of death registration in Ghana using demographic and statistical techniques. These methods produced varied completeness estimates, ranging from 28 percent to 56 percent, indicating that death registration in Ghana remains low and incomplete, with little improvement in the last two decades. The findings have considerable implications for implementing effective public health policy; without accurate mortality data, it will be difficult to understand the disease burden and identify high-risk populations. Low completeness rates can lead to misallocation of resources aimed at reducing the burden of mortality at lower levels of administration. Given that Greater Accra is the most urbanized and least poor region, the level of completeness is far below the acceptable 80 percent threshold, as measured by using both the empirical completeness and death distribution methods. In addition, the below 30 percent completeness recorded in most regions is concerning; hence, strategic and tailored regional interventions must be prioritized. Government programs to improve completeness of civil registration should not prioritize birth registration at the expense of death registration. There is a need for concentrated efforts to improve Ghana’s CVRS. Policies and programs are needed to raise awareness on the importance of death registration, particularly in districts farther away from the administrative centre. To achieve near-completeness of death registration, resources need to be marshalled in both the formal and non-formal systems if Ghana is to improve its death registration system. Additionally, existing legal mechanisms should be enforced: while in Ghana, cemeteries are required to request a death certificate before permitting a burial, this policy is inconsistently enforced, particularly in rural and peri-urban areas. Strengthening enforcement of this legal provision and extending it uniformly across cemetery types, including those managed by private entities, community groups, and religious bodies, could serve as a low-cost lever to increase registration rates by creating a practical incentive to register deaths before burial.

## Supporting information

S1 Table Estimate of completeness from empirical completeness model. (DOCX)

S2 Table Mortality information data silos in Ghana. (DOCX)
