# Supplementary material for: Completeness of death registration in Ghana: An evaluation of multiple data sources and methods
Source: PLoS One. 2026 Jul 23;21(7):e0354361. doi: 10.1371/journal.pone.0354361 (PMC13395453; doi:10.1371/journal.pone.0354361)
Supplement: S1 Table — (DOCX) [file pone.0354361.s001.docx]

S1 Table - Estimate of completeness from empirical completeness model

| Region | Empirical model 1 | | | Empirical model 2 | | |
| --- | --- | --- | --- | --- | --- | --- |
|  | Both | Male | Female | Both | Male | Female |
| Ghana | 15.2 | 16.8 | 19.2 | 28.7 | 28.9 | 31.6 |
| Western | 9.6 | 10.8 | 11.7 | 17.1 | 18.1 | 18.6 |
| Central | 11.8 | 10.9 | 18.7 | 21.5 | 18.0 | 31.2 |
| Greater Accra | 42.6 | 41.0 | 64.3 | 69.8 | 62.0 | 81.0 |
| Volta | 24.5 | 30.0 | 25.5 | 47.1 | 52.9 | 43.9 |
| Eastern | 21.1 | 24.3 | 25.0 | 39.0 | 40.6 | 39.9 |
| Ashanti | 15.7 | 19.5 | 17.0 | 29.9 | 34.4 | 28.1 |
| Western North | 13.0 | 10.8 | 25.8 | 25.3 | 18.6 | 45.0 |
| Ahafo | 15.6 | 19.7 | 17.9 | 29.3 | 33.5 | 29.0 |
| Bono | 15.8 | 14.7 | 26.8 | 29.8 | 25.0 | 44.0 |
| Bono East | 7.3 | 8.4 | 8.8 | 12.3 | 13.6 | 13.4 |
| Oti | 5.1 | 5.2 | 6.7 | 7.6 | 7.5 | 9.6 |
| Northern | 9.1 | 10.8 | 10.8 | 16.3 | 18.6 | 17.3 |
| Savannah | 6.7 | 6.9 | 9.1 | 11.1 | 10.8 | 14.1 |
| North East | 12.8 | 42.3 | 16.4 | 22.8 | 59.7 | 24.9 |
| Upper East | 5.9 | 8.4 | 5.0 | 9.2 | 13.7 | 6.8 |
| Upper West | 7.8 | 7.4 | 13.8 | 13.1 | 11.4 | 23.0 |
