# Supplementary material for: Completeness of death registration in Ghana: An evaluation of multiple data sources and methods
Source: PLoS One. 2026 Jul 23;21(7):e0354361. doi: 10.1371/journal.pone.0354361 (PMC13395453; doi:10.1371/journal.pone.0354361)
Supplement: S2 Table — (DOCX) [file pone.0354361.s002.docx]

S2 Table – Mortality information data silos in Ghana.

| **Name** | **Level** | **Description** |
| --- | --- | --- |
| Family | Local | The beneficiary of the system |
| Health facility | Local | Medical certification of the fact and cause of death |
| Police | Local | All deaths outside a health facility |
| Coroner | Local | All deaths outside a health facility |
| Mortuary | Local | Facility in national and regional hospitals |
| Pathologists | Local | A scientist who studies the causes and treatment of diseases |
| Funeral homes | Local | Burial |
| Cemetery | Local | Burial |
| Religious bodies (Church / Mosque) | Local | Burial |
| Births and Deaths Registry (Local offices) | Local | Registration of deaths |
| Health and Demographic Surveillance Sites (HDSS) | Local | Deaths in surveillance sites |
| Demographic and Health Surveys | National | Aggregated mortality for survey deaths |
| Population and Housing Census | National | Aggregated mortality for all survey-reported deaths |
| Ghana Health Service | National | Aggregated mortality statistics for all deaths in a health facility |
| Judiciary system | National | All deaths outside the health facility |
| Births and Deaths Registry (National) | National | Aggregated mortality for all registered deaths |
| Adapted from [1] |  |  |

1. Cobos Muñoz D, Sant Fruchtman C, Miki J, Vargas-Herrera J, Woode S, Dake FAA, et al. The Need to Address Fragmentation and Silos in Mortality Information Systems: The Case of Ghana and Peru. International Journal of Public Health. 2022;67: 1–9. doi:10.3389/ijph.2022.1604721
